# Supplementary material for: An oscillating computational model can track pseudo-rhythmic speech by using linguistic predictions
Source: eLife. 2021 Aug 2;10:e68066. doi: 10.7554/eLife.68066 (PMC8328513; doi:10.7554/eLife.68066)
Supplement: Supplementary file 1. [file elife-68066-supp1.docx]

**Supplementary Information**

**Supplementary tables**

| **Supplementary table 1.** Summary of regression model for logarithm of word duration | | | | | | | |
| --- | --- | --- | --- | --- | --- | --- | --- |
| **Variable** | **Trans** | **B** | **β** | **SE** | **t** | **p** | **VIF** |
| Intercept | x | -0.2612 |  | 0.054 | -4.873 | 0.009 |  |
| RNN prediction | x ^(1/6)^ | 0.0504 | 0.0148 | 0.037 | 1.346 | 0.178 | 1.5 |
| Bigram | log(x) | 0.0432 | 0.1420 | 0.004 | 10.790 | <0.001 | 2.2 |
| Word frequency | x | 0.0843 | 0.1552 | 0.007 | 11.949 | <0.001 | 2.1 |
| Mean duration | log(x) | 1.0595 | 0.7657 | 0.019 | 55.905 | <0.001 | 2.4 |
| Syllable Rate | x | -0.0440 | -0.1113 | 0.004 | -12.466 | <0.001 | 1.0 |
| Model R^2^ = 0.558. Trans = transformation, B = unstandardized  coefficient, β = standardized coefficient, SE = standard error, t = t value, p = p value,  VIF = variance inflation factor | | | | | | | |
